# Coress feedback

**DOI:** 10.1308/003588414X13814021678475

**Published:** 2014-01

## Abstract

CORESS is a confidential reporting system for surgery. The purpose of CORESS is to promote safety in surgical practice, both within the NHS and in the independent sector.

In a deviation from normal practice, the clinical emphasis in this issue of CORESS Feedback has been provided by the Clinical Board for Surgical Safety of the Royal College of Surgeons, at the behest of the National Patient Safety Agency (NPSA), which wishes to draw the attention of surgeons to the risks of inadvertent removal of tissue of other histological origin when operating on pregnant women for presumed appendicitis. Publication of the vignettes below illustrates a sample from a larger series of similar cases reported to the NPSA and represents collaboration between our organisations, for which education of surgeons and improvement of patient safety are common goals.

We are grateful to the clinicians who have provided the material for these reports. The online reporting form is on our website (www.coress.org.uk), which also includes all previous Feedback Reports. Published contributions will be acknowledged by a ‘Certificate of Contribution’, which may be included in the contributor’s record of continuing professional development.

## Ovarian tissue mistaken for appendix (case 1) (Ref 157)

An 11-year-old girl presented to the emergency department with abdominal pain in the early hours of the morning. She was reviewed in the department, referred to the paediatric team and admitted to the children’s ward, where she was seen by the surgical registrar, who indicated the likely diagnosis was appendicitis.

The following morning (a weekend) the child was seen by the consultant surgeon on his post-take ward round. He explained to the child’s mother that although the clinical picture was not ‘classic for appendicitis’, it was recommended to proceed to an appendicectomy.

The surgery was carried out that afternoon by a locum registrar; this was this registrar’s first operation in the hospital. The operating registrar was the senior surgical doctor in the operating theatre. The consultant attended the operating theatre, enquiring during the surgery whether there were any problems, and was told by the operating registrar that the appendix had been removed. On viewing the removed tissue, the consultant thought it looked slightly smaller than would be expected. The operation note in the medical record describes the removed tissue as an ‘inflamed retrocaecal 3cm appendix’ and states there was turbid peritoneal fluid.

The child made an uneventful recovery but three days later the consultant histopathologist reported to the consultant surgeon that the tissue removed was prepubertal ovarian tissue and that no appendiceal tissue was present. Investigations confirmed there were no errors in labelling of the specimen in theatre and subsequent deoxyribonucleic acid testing proved the ovarian tissue to be that of the child in question. The parents were contacted by the consultant surgeon as soon as the error was discovered and a full explanation was given.

**Contributory factors as identified by the trust:**
- There was an absence of any written guidelines on paediatric surgery in the trust.
- The locum registrar was filling a vacancy left by a middle grade doctor leaving the trust and prior to the replacement commencing.

Following this incident, the trust developed protocols and a hierarchy of responsibility in line with the national and regional guidelines for paediatric leads. The trust stated that children should usually be operated on by the consultant or under direct consultant supervision.

This incident was discussed at the NPSA’s response meeting as a ‘never event’ and a letter was sent to the trust’s medical director in line with the processes in place at the time. The response included details of the investigation as summarised above.

## CORESS comments

The CORESS Advisory Committee drew attention to the responsibilities of the trust to ensure certified competence of employees for the roles in which they are employed. It was felt that the consultant who takes responsibility for the patient has a duty to satisfy himself or herself of the operator’s competence and that in this case, the consultant should have been supervising in a scrubbed capacity.

## Fallopian tube mistaken for appendix (case 2) (Ref 158)

A 28-year-old patient, who was 15 weeks pregnant, presented to the emergency department with low abdominal and acute iliac fossa pain. A diagnosis of acute appendicitis was made by the surgical registrar and this was discussed with the consultant surgeon. Arrangements were made for an open appendicectomy to be carried out by the registrar. The consultant was happy for the registrar to undertake the surgery as he was in a non-training post and had been operating independently for some months. Surgery was undertaken late at night, at the end of a prolonged on-call period. There was no first assistant at the operation and the scrub nurse performed the dual role of assistant as well as scrub nurse.

The surgical findings were recorded as a mildly inflamed, very long appendix adherent to the right ovary. An inflamed structure lay between the pregnant uterus and caecum. The mesoappendix was ligated and divided. The appendix stump was tied but not buried. The right ovary was large and contained a cyst. The gynaecology registrar was asked to attend and give an opinion. By the time he came, the appendix was already separate from the ovary so he only saw the ovary. The opinion was that the appearance of the ovary was consistent with pregnancy.

The patient made a good recovery and was discharged on the second postoperative day. Four days following surgery, a consultant histopathologist contacted the on-call surgical registrar to advise that the structure removed at operation was not an appendix but a fallopian tube. The consultant then arranged to see the patient in the outpatient clinic and the situation was explained to her fully. A laparoscopic appendicectomy was recommended following the delivery of her baby.

**Root causes as identified by the trust:**
- A protocol regarding women presenting to the emergency department in early pregnancy with emergency abdominal pains for joint surgical/obstetrics and gynaecology assessment was not widely known or followed.
- There was a practice within the specialty to work an unacceptable on-call pattern. There was a breach of the European Working Time Regulations regarding adequate rest.
- The role of scrub nurse in theatres was variable. Acting as first assistant is not part of the job description; additional support for this is based on goodwill and level of competence.
- Incident reporting culture and awareness among certain staff groups was poor. There was lack of clarity regarding the process and mechanisms that can be triggered and accessed via this route, which would have ensured that the patient was informed in a more timely and supportive manner as well as providing support for the registrar/consultant.

## CORESS comments

The CORESS Advisory Committee drew attention to the fact that, where possible, such operations should not be carried out semielectively at night. It was felt that the role of the scrub nurse as assistant was not a specific contributory factor in this case and that it was acceptable for an experienced nurse to act in this capacity in these circumstances.

## Fallopian tube mistaken for appendix (case 3) (Ref 159)

A 34-year-old patient, who was 17 weeks pregnant, was admitted to hospital with lower abdominal pain. After review by the obstetric registrar, she was referred to the surgical team. The next morning, following the registrar ward round and discussion with the consultant surgeon on call, ultrasonography was carried out, confirming the diagnosis of acute appendicitis. The registrar discussed the results with the consultant and it was agreed that an appendicectomy should be undertaken. The patient refused this option initially despite the explanation by the registrar of the risks to herself and her unborn baby. Consequently, a conservative management plan with intravenous antibiotics was commenced but later that day, the patient relented and consented to undergo surgery.

The consultant was happy for the registrar to undertake the procedure and confident in his ability to perform open appendicectomy unsupervised. The surgery took place that evening and postoperative recovery was uneventful. Postoperatively, it was discovered that the pathology results for the excised tissue were inconsistent with the clinical picture, eliciting an urgent telephone call to the consultant. The patient was contacted and met with the consultant surgeon to discuss the outcome of the surgery. Appropriate arrangements were made for follow-up care in line with the trust’s ‘being open’ policy. The incident was investigated internally and an external surgical review was undertaken by the local deanery

The registrar’s clinical knowledge, skills and experience were reviewed independently and it was concluded that he had the knowledge, skills and experience to have been carrying out this surgical procedure. Furthermore, his logbook demonstrated evidence of assessed competency in carrying out this procedure on a number of occasions, including three cases where there were pregnancies. On interview, the registrar was able to provide clear and concise recollection of the procedure, and he was confident at the time that the structure he removed was the appendix. He recalls demonstrating the anatomy to the foundation trainee as he proceeded and commented that he did not recall seeing any fimbriae. It was concluded that it was reasonable for the registrar, with assessed competence to undertake this level of surgical procedure, to proceed unsupervised.

Following the incident, the registrar was relieved of emergency care duties and operating in any capacity until the outcome of the investigation was known. The programme director (regional) arranged for a period of training to be undertaken over the next 6–12 months in another acute trust under the guidance of a consultant surgeon, with clear objectives agreed.

**Root cause as determined by the trust:**
- It was concluded that this incident was the result of human error.

## CORESS comments

These salutary cases are among a number of similar cases received by the NPSA. The Clinical Board for Surgical Safety has been directed by the NHS Commissioning Board to set up a ‘never events’ task force to reduce (eradicate) such incidents. CORESS is represented on this task force and supports the initiative to reduce the incidence of adverse events such as those described above. The problems of appropriate assessment, disorientation due to disordered anatomy and failure to request help when unsure of one’s ability to make safe progress remain common themes in reported incidents. Readers contemplating an appendicectomy in a pregnant woman or female child should consider the lessons arising from these cases.

